# Effects of cropping systems upon the three-dimensional architecture of soil systems are modulated by texture

**DOI:** 10.1016/j.geoderma.2018.07.002

**Published:** 2018-12-15

**Authors:** Aurelie Bacq-Labreuil, John Crawford, Sacha J. Mooney, Andrew L. Neal, Elsy Akkari, Cormac McAuliffe, Xiaoxian Zhang, Marc Redmile-Gordon, Karl Ritz

**Affiliations:** a Division of Agriculture & Environmental Sciences, School of Biosciences, University of Nottingham, Sutton Bonington Campus, Leicestershire LE12 5RD, UK; b Sustainable Agriculture Science, Rothamsted Research, West Common, Harpenden, AL5 2JQ, UK

**Keywords:** X-ray CT, Cropping systems, 3D image analysis, Porosity, Pore size distribution, Pore connectivity

## Abstract

Soil delivers fundamental ecosystem functions via interactions between physical and biological processes mediated by soil structure. The structure of soil is also dynamic and modified by natural factors and management intervention. The aim of this study was to investigate the effects of different cropping systems on soil structure at contrasting spatial scales. Three systems were studied in replicated plot field experiments involving varying degrees of plant-derived inputs to the soil, viz. perennial (grassland), annual (arable), and no-plant control (bare fallow), associated with two contrasting soil textures (clayey and sandy). We hypothesized the presence of plants results in a greater range (diversity) of pore sizes and that perennial cropping systems invoke greater structural heterogeneity. Accordingly, the nature of the pore systems was visualised and quantified in 3D by X-ray Computed Tomography at the mm and μm scale. Plants did not affect the porosity of clay soil at the mm scale, but at the μm scale, annual and perennial plant cover resulted in significantly increased porosity, a wider range of pore sizes and greater connectivity compared to bare fallow soil. However, the opposite occurred in the sandy soil, where plants decreased the porosity and pore connectivity at the mm scale but had no significant structural effect at the μm scale. These data reveal profound effects of different agricultural management systems upon soil structural modification, which are strongly modulated by the extent of plant presence and also contingent on the inherent texture of the soil.

## Highlights

• Degree of plant presence in agricultural systems profoundly affects soil structure.
• Such effects are contingent on the soil texture.
• Systems involving plants invoke greater porosity and pore size range in clay soil.
• Systems involving plants invoke reduced porosity and permeability in sandy soil.

## 1 Introduction

Soil structure is dynamic and subject to modification by natural and anthropogenic actions, such as wetting-drying cycles and freeze-thaw action. These processes re-structure the soil with potential consequences for physical and biological processes (Rabot et al., 2018). Water flow and gas diffusion are both affected by the porous architecture (Naveed et al., 2016). The nature and magnitude of soil microbial activity are affected by the air-water balance in soil and the availability of nutrients, and microbial communities are strongly affected by their microenvironment in soil (Chenu, 1993; Helliwell et al., 2014). Soil microbes, along with plant roots, are implicated in aggregation processes via gluing and enmeshing activity (Tisdall and Oades, 1982). Microbial communities can contribute to aggregate stability and therefore help prevent de-structuring of soil structure (Chenu and Cosentino, 2011; Dorioz et al., 1993; Oades, 1993). This, in turn, might lead to the capacity of soils to adapt to changing environmental circumstances (Crawford et al., 2012; Feeney et al., 2006).

Tillage practices have a significant direct influence impact on soil structure, often increasing the macro-porosity of conventionally managed soils (Ambert-Sanchez et al., 2016). Conventional tillage can also result in depletion of nutrients and organic carbon within the soil (Coleman et al., 1997) and a decline in aggregated structure (Watts et al., 2001). Studies of a long-term (40+ years) field experiment at Rothamsted Research (Harpenden, UK) in which grassland was converted to arable and bare fallow managements has resulted in a decline of soil organic carbon and nitrogen (Gregory et al., 2016) and a decrease in microbial abundance under the different managements (Hirsch et al., 2009). These studies focused on soil biological and chemical properties (such as microbiota, pH, organic carbon). However, there is no information on the structure of the pore networks of the soils under these long-term managements.

The aim of this study was to identify any effects of different cropping managements associated with contrasting degrees of plant presence on soil structure in the context of two soil textural classes. Three long-term field cropping systems were studied: grassland (perennial plant), arable (annual plant) and bare fallow (no plant). We hypothesized that cropping management influences the inherent soil structural properties by (i) the presence of plants resulting in greater soil porosity and range of soil pore diameters due to root action; and (ii) a more persistent presence of plants invokes greater porosity and structural heterogeneity, apparent as a wider range of pore sizes in perennial systems. Structural properties of the soils were determined at two spatial scales of sample and resolution, i.e. ‘core’ scale (435 cm^3^ at 40 μm resolution) and ‘aggregate’ scale (circa 4 mm^3^ at 1.5 μm resolution). To establish the functional consequences of such structures for water flow in the soils, we also estimated their saturated hydraulic conductivity at both scales using a pore-scale modelling approach.

## 2 Materials and methods

### 2.1 Soils

Soil cores were collected in October 2015 at Rothamsted Research (Hertfordshire, UK) from two complementary long-term experiments: Highfield Ley-Arable experiment (LATLONG 51.8103N, -0.3748E), on a silty-clay loam textured soil developed on clay-with-flints over Eocene London Clay (Batcome series) and classified as a Chromic Luvisol by FAO criteria (hereafter referred to as clay soil, Table 1); and Woburn Ley-Arable experiment (LATLONG 52.0009N, -0.6137E), on a well-draining, sandy loam soil of the Cottenham Series (Hodge et al., 1984), classified as a Cambric Arenosol (FAO), (referred to as sandy soil, Table 1). The replication of the treatments was uneven and based on the inherent experimental plot design. Four cylindrical cores (68 mm diameter × 120 mm height) of the grassland and arable treatments and three replicate cores of the bare fallow treatment were extracted for the clay soil from the surface down to the height of the columns minus 1 cm (110 mm). Four cores of the grassland, arable with manure (25 t ha^−1^ annum^−1^; hereafter referred to as arable manure) and bare fallow treatments and five replicate cores of the arable with inorganic fertiliser (10 kg N ha^−1^ y^−1^; hereafter referred to as arable inorganic) were collected for the sandy soil. For both soil types, the arable treatment was under conventional tillage, ploughed to a depth of 23 cm, once a year. The arable fields for the clay and sandy soil was last ploughed respectively in September and October 2014 before sampling. The fallow plots were rotavated in June 2015 for the clay soil and tined in April 2015 for the sandy soil before sampling. All replicates were independent and derived from separate plots. All treatments had been maintained for at least 50 years. After sampling, cores were stored at 4 °C prior to further analysis.

**Table 1:** Summary physical and chemical data of Highfield Ley-Arable experiment soils.

Treatment	Density^a^/g cm^−3^	pH^a^ (H_2_O)/-log(g[H^+^]L^−1^)	Organic carbon^a^/mg g^−1^ soil	Free organic carbon^b^/μg g^−1^ soil	Intra-aggregate organic carbon^b^/μg g^−1^ soil	Nitrogen^a^/μg g^−1^ soil	NaOH-EDTA extractable phosphorus^c^/μg g^−1^ soil
Fallow	1.30–1.45	5.1	0.8	150	380	100	235
Arable	1.30–1.45	5.8	1.3	370	490	150	517
Grassland	0.99	6.0	3.9	4690	3010	390	662

a Gregory et al., 2016.

b Hirsch et al., 2009.

c Neal et al., 2017.

### 2.2 X-ray computed tomography (CT)

Soil cores were scanned using a Phoenix v∣tome∣x M scanner (GE Measurement and Control solution, Wunstorf, Germany), set at 160 kV, a current of 180 μA, detector sensitivity of 200% and at a pixel/voxel resolution of 40 μm (resultant voxel volume = 64,000 μm^3^). A total of 2900 projection images were taken at 250 ms per image using an averaging of 1 image and skip of 0. Total scan time per core was 24 min. After scanning, each core was dismantled, and the soil passed through a sieve series of 4, 2 and 0.71 mm. Three randomly-selected aggregates retained between the 2 and 0.71 mm sieves per core were scanned using a Phoenix Nanotom® (GE Measurement and Control solution, Wunstorf, Germany) set at 90 kV, a current of 65 μA and at a base resolution of 1.51 μm (resultant voxel volume = 3.44 μm^3^). A total of 1440 projection images were taken at 500 ms period using an averaging of 3 images and skip of 2. The total scan time per sample was 69 min.

Reconstruction of all scanned images was processed using Phoenix datos∣x2 rec reconstruction software. Scanned images were optimised to correct for any movement of the sample during the scan and noise was reduced using the beam hardening correction algorithm, set at 8. As a multi-scan routine was performed on the core samples, VG StudioMax® 2.2 was used to merge the top and bottom scans to obtain a single 3D volume for the complete core. For both core and aggregate samples, image sequences were extracted (dimensions described below) for image analysis. Core samples were scanned at the prevailing water content following sampling (approximately field capacity). Soil aggregates were derived from these cores following air-drying overnight and the moisture content recorded. The soil was passed through 4, 2 and 0.71 mm mesh size sieves while subjected to horizontal shaking for 3 min at 300 rotations min^−1^. Twenty aggregates were randomly selected from between the 2 and 0.71 mm sieves, and conserved in sealed containers in the dark at room temperature.

### 2.3 Image analysis

Initial image analysis was performed using ImageJ (Schneider et al., 2012). For both soil cores and aggregates, a uniform region of interest (ROI) was defined for each sample; 40 × 40 × 40 mm and 0.981 × 0.725 × 0.604 mm respectively. Core ROIs were positioned centrally to limit inclusion of cracks or large stones created during the sampling process. Cubic ROIs for aggregates were not possible because of their variable geometry, so the largest ROI accommodated by all aggregates was chosen. The coordinates of these regions were adapted for each image volume/sequence. The image pre-processing consisted of: (i) cropping to the ROI; (ii) enhancing the contrast/brightness to 0.35%; (iii) application of a 2-pixel radius median filter; (iv) converting the image format to 8-bit; (v) saving the new image volume. Stones were segmented from the ROI volume in VG StudioMax® 2.2 using the surface determination tool.

All images were thresholded using the bin bi-level threshold approach by Vogel and Kretzschmar (1996) using the open source software QuantIm (http://www.quantim.ufz.de/). Each image within the image sequence has a single threshold value, to determine the prescribed initial threshold values (T_1_ and T_2_), the Li-threshold algorithm in ImageJ was applied to 20 images randomly selected within the image sequence. The threshold values (T_1_ and T_2_) were attributed depending on extremes values obtained in the 20 images selected. Porosity, pore size distribution, pore connectivity and surface density determined according to Vogel et al. (2010). Here, total porosity refers to percentage of pores > 0.05 mm at the core scale and >1.8 μm at the aggregate scale, as per the segmented data. Pore size distribution was expressed as the proportion of each pore size as the percentage volume normalised to the total ROI volume, and also as the proportion of each pore size as the cumulative percentage volume normalised to the total pore volume. The pore size diameter was determined using a maximum opening diameter based on a numerical sphere algorithm as described by Vogel et al. (2010). Pore connectivity is derived from the Euler number and normalised by the total volume. The more negative the Euler number, the more connected the pore system. Increase of sphere opening diameter results in loss of smaller pores or pore-irregularities due to irregular-shape pores. The surface density represents the proportion of transition from pore to solid which is the ratio of the pore surface area (mm^2^) divided by the pore volume (mm^3^). After the spherical opening, the numerical representation of the pore surface includes a combination of the pore-solid interface and a numerical surface placed in the middle of irregular-shaped pores (Vogel et al., 2010).

### 2.4 Hydraulic conductivity of aggregates and inter-aggregate pores

It is not straightforward to measure saturated hydraulic conductivity (*K*_sat_) of individual aggregates. To facilitate direct comparison of *K*_sat_ for all sample classes, we numerically calculated the ability of inter- and intra-aggregate pores to conduct water based on the pore-scale velocity of water flow through the pore geometry. This was simulated using the lattice Boltzmann model developed previously (Zhang et al., 2005, Zhang et al., 2016; Zhang and Lv, 2007). The details of the model and how the permeability of each image was calculated are given in Appendix A.1. Supplementary materials. The computational demand of the ROI exceeded the power of the computer facility we could access, and we were therefore unable to directly simulate water flow in the ROI. Instead, we selected several sub-volumes, denoted as volume of interest (VOI), from each ROI to simulate water flow through them and calculated their permeability. The model used a sparse matrix algorithm to store only the pore voxels, and hence the maximum size of the VOI the model can process depended on the number of pore voxels (or porosity) within it. For each ROI image, we first calculated its porosity and then divided it into a number of equal sub-volumes ensuring that the number of pore voxels in each sub-volume did not exceed the maximum pore voxels the model can deal with. Pre-analysis of all images revealed that the pore geometry in the aggregates was a relatively uniform 0.5 mm, we thus used one VOI (375 ∗ 375 ∗ 375 μm) to represent one ROI. The pore geometry in the core was highly heterogeneous, and we randomly selected three samples from the sub-volumes into which the original ROI was divided to simulate water flow and calculate their permeability.

### 2.5 Statistical analysis

Analysis of variance (ANOVA) was performed on all primary variables using a split-plot design with cropping management and size classes of pores as factors. The relationship between the measured porosity and calculated saturated permeability was also explored using ANOVA to test for equality of slopes of log_10_-transformed data within the modelled VOI. All analyses were conducted using Genstat v 17.1 (VSN International Ltd. 2014).

## 3 Results

### 3.1 Effect of management on clay soil pore structure

#### 3.1.1 3D image assessment

**Fig. 1:**
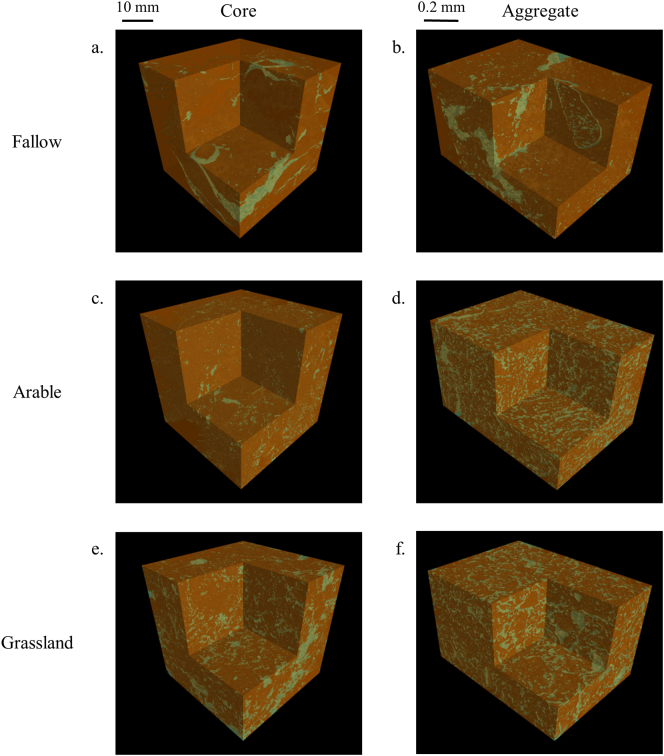
3D representation of clay soils under different cropping systems visualised at core (40 μm resolution; a, c, e) and aggregate (1.5 μm resolution; b, d, f) scales, displayed as thresholded images denoting pore (green) or solid (brown) phases. (a, b) bare fallow; (c, d) arable; (e, f) grassland. (For interpretation of the references to colour in this figure legend, the reader is referred to the web version of this article.)

**Fig. 2:**
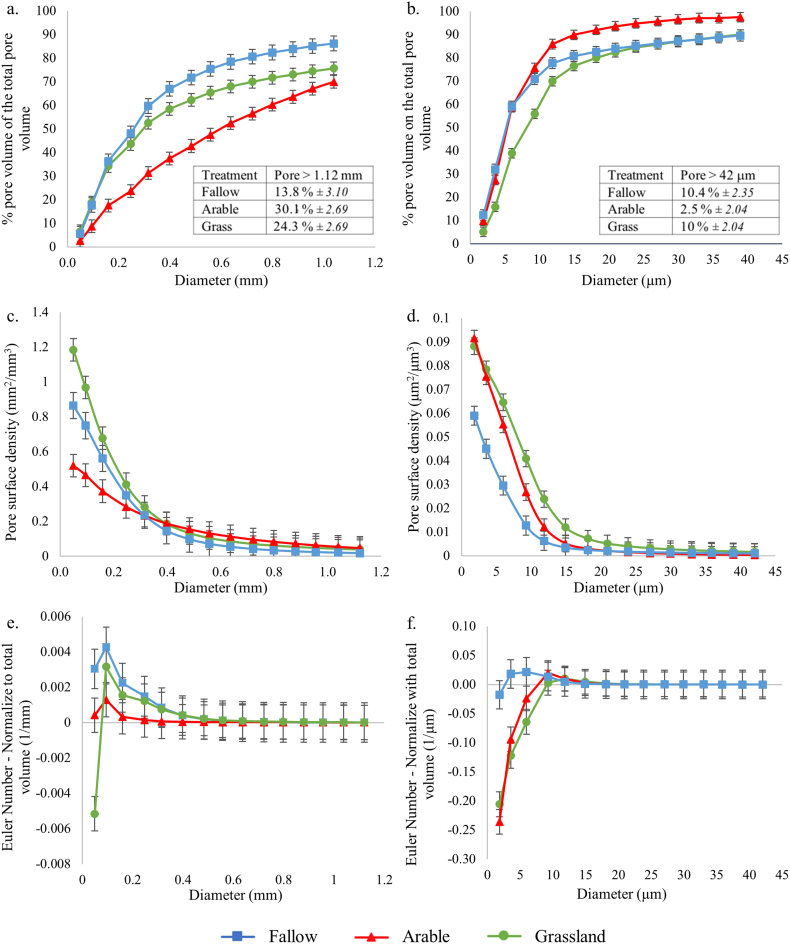
Soil pore characteristics of clay soils under different cropping systems at core (40 μm resolution; a, c) and aggregate (1.5 μm resolution; b, d, e) scales: (a, b) cumulative pore distribution of cores; (c, d) surface density; (e, f) connectivity. Points indicate means, whiskers denote pooled standard errors.

Fig. 1 illustrates selected 3D representations of pore architecture from the clay soil cores (Fig. 1a, c, e) and aggregates (Fig. 1b, d, f) displayed as segmented images. For the soil cores, there was a clear decrease in the number of stones respectively fallow = arable > grass (Fig. A1, Table A1). Moreover, arable soils contained larger pores (>1 mm) than the other treatments (Fig. 1c), especially at the interface with stone material (Fig.1c). The bare fallow core generally had smaller pores (0.25–1 mm; Fig. 1a). Grass cores had a wider range of pore sizes and contained more root and organic material (Fig. 2a, A1e). A similar observation was made for soil aggregate images as the bare fallow aggregates comprised mostly small pores despite the presence of a few, larger pores (Fig. 1b, A1b), the arable aggregates appeared to only have smaller sized pores (Fig. 1d, A1d), and grass aggregates again showed the widest range of pore sizes (Fig. 1f, A1f).

#### 3.1.2 3D characteristic analysis

There were significant differences in porosity characteristics for the clay soil under each management system, which contrasted in nature between core and aggregate scales (Table 2; Fig. 1). At the core scale, total pore volume was not significantly different between treatments (Table 2). At the aggregate scale, total pore volume was significantly different under all three treatments, with a distinct ranking of grassland > arable > fallow, and a two-fold difference between grassland and fallow (Table 2).

**Table 2:** Total porosity in relation to management type at the core (base resolution 40 μm) and aggregate (base resolution 1.5 μm) scale of the clay soil, expressed as percentage of pores relative to the total volume (mean ± pooled standard error).

Treatment	*n*	Core	Aggregate
Fallow	3	8.07 (±0.76)	14.3 (±1.08)
Arable	4	8.29 (±0.66)	23.4 (±0.94)
Grassland	4	12.0 (±0.66)	31.1 (±0.94)
*P*_F_		0.53	<0.001

The pore size distribution normalised to the total ROI volume showed, at the core scale, for the arable treatment an equivalent proportion of all pore sizes < 1.12 mm, with a smaller proportion of pore sizes < 0.25 mm compared to grassland and fallow treatments (Fig. A2a). The proportion of pores > 1.12 mm was greater for the grassland and the arable treatments compared to fallow treatment (Fig. A2a). Cumulative pore size distributions were similar in their non-linear character under grassland and fallow at the core scale, showing a greater proportion of pore sizes < 0.25 mm than the arable soil (i.e. >50% of pore volume; Fig. 2a). However, the proportion of pore sizes with a diameter > 1.12 mm was significantly greater under grassland and arable than fallow (*P* < 0.001; Fig. 2a). In contrast, under the arable treatment, the cumulative pore size distribution was linear between 0.05 and 1.04 mm (Fig. 2a), indicating that the pores in this range were uniformly distributed. At the aggregate scale, the proportion of pore sizes normalised to the total ROI volume increased significantly with the increasing pore sizes up to 5.97 μm for all treatments with a greater proportion of these pore sizes for grassland and arable treatments. For the pore sizes between 9.26 and 20.91 μm, the proportion of the pore sizes decreased for all treatment, with a substantial decrease for the arable compared to the grassland. There were no significant differences beyond this pore size, except for the pore sizes > 42 μm with the proportion of pores ranking from arable < fallow < grassland (Fig. A2b). The cumulative pore size distributions for pores < 9.26 μm under arable and fallow treatments were not significantly different, but both were significantly greater than grassland (Fig. 2b). For pores > 11.8 μm, the relationship was reversed and distributions under grassland and fallow were not significantly different, but arable had a significantly smaller proportion of larger pores (Fig. 2b).

At the core scale, the pore-surface density was significantly different under all three treatments for pore sizes < 0.095 mm, with ranking of arable < fallow < grassland (Fig. 2c), with a two-fold difference between grassland and arable. For the pore sizes < 0.25 mm, the surface density declined similarly under grassland and arable and there was no difference beyond the pore size 0.31 mm under all three treatments (*P* *<* 0.001; Fig. 2c). In contrast, at the aggregate scale, the surface density relating to the smallest pore sizes (1.86 μm) was significantly reduced under fallow compared to arable and grassland, which were not significantly different (*P* *<* 0.001; Fig. 2d). However, for the pore sizes between the pores 5.97 μm and 11.8 μm, the surface density decreased more drastically under the arable treatment compared to the grassland, both converging towards the fallow. The surface density was not significantly different for pores > 14.9 μm under all treatments (Fig. 2d).

There were no significant differences in pore connectivity between any of the treatments at the core scale (*P* *>* 0.05; Fig. 2e). At the aggregate scale, the connectivity was significantly greater under grassland and arable treatments than fallow with respect to pores < 5.97 μm, with no differences beyond this (Fig. 2f). There was no change in pore connectivity within aggregates under fallow across the size range measured (Fig. 2f).

At the core scale, soil porosity within the VOI used for permeability simulation was linearly related to porosity of ROI (*P* < 0.001), but was on average 73% greater (*P* < 0.001; data not shown), indicating that the pore geometry in ROI was highly heterogeneous at centimetre scale. Within VOI, porosity was significantly greater under grassland than fallow, with arable intermediate but not significantly different from either (Table 4). Simulated permeability mirrored these trends, and was circa two-fold greater for grassland than fallow (Table 3). There was a significant positive power-law relationship between porosity and permeability in the case of fallow and arable, and marginally so for grassland (Fig. A3a). Across all three treatments there was no significant difference between the regression coefficients for the power-law relationships (overall mean 1.12 ± 0.30; Table 4). At the aggregate scale, the porosity of VOIs and ROIs of aggregates was not different (*P* < 0.001; data not shown), revealing that at 0.3–0.5 mm scale the aggregates were relatively uniform. Here, mean porosity was significantly different between all treatments in the rank order of grassland > arable > fallow (Table 3). Modelled permeability in grassland treatments was double that in arable and fallow, which were not significantly different from each other (Table 3). At this scale, there was a significant positive power-law relationship between porosity and permeability in all cases which was weakest for fallow (Fig. A3b), but as for the core-scale, there was no significant difference between the regression coefficients across all treatments (Table 4; overall mean 1.0 ± 0.23).

**Table 3:** Total porosity in relation to management type (expressed as percentage of pores relative to the total volume), and modelled saturated permeability, of the volume of interest used for modelling, for the clay soil at the core and aggregate scale (mean ± pooled standard error).

Treatment	*n*	Core		Aggregate	
		Porosity (%)	Permeability (mm^2^)	Porosity (%)	Permeability (mm^2^)
Fallow	3	9.3 (±2.32)	387 (±202)	14.8 (±1.78)	0.55 (±0.09)
Arable	4	12.0 (±2.01)	702 (±175)	23.0 (±1.54)	0.62 (±0.08)
Grassland	4	16.3 (±2.01)	827 (±175)	31.0 (±1.54)	1.13 (±0.08)
*P*_F_		0.54	0.38	0.002	0.003

**Table 4:** Linear regression coefficients (mean ± standard error) in relation to management type of log porosity vs. log modelled saturated permeability for the clay soil, at the core (Fig. A3a) and aggregate scale (Fig. A3b).

Treatment	*n*	Core		Aggregate	
		Coefficient^a^	*P*_uncorr_	Coefficient^a^	*P*_uncorr_
Fallow	9	0.27 (±0.13)	0.08	0.71 (±0.24)	0.02
Arable	12	0.79 (±0.07)	<0.001	0.93 (±0.14)	<0.001
Grassland	12	1.00 (±0.15)	<0.001	1.38 (±0.32)	0.002
Coefficients P_*F*_		0.01		0.30	

a Coefficient of x value in fitted equation.

### 3.2 Effect of management on sandy soil

#### 3.2.1 3D image assessment

**Fig. 3:**
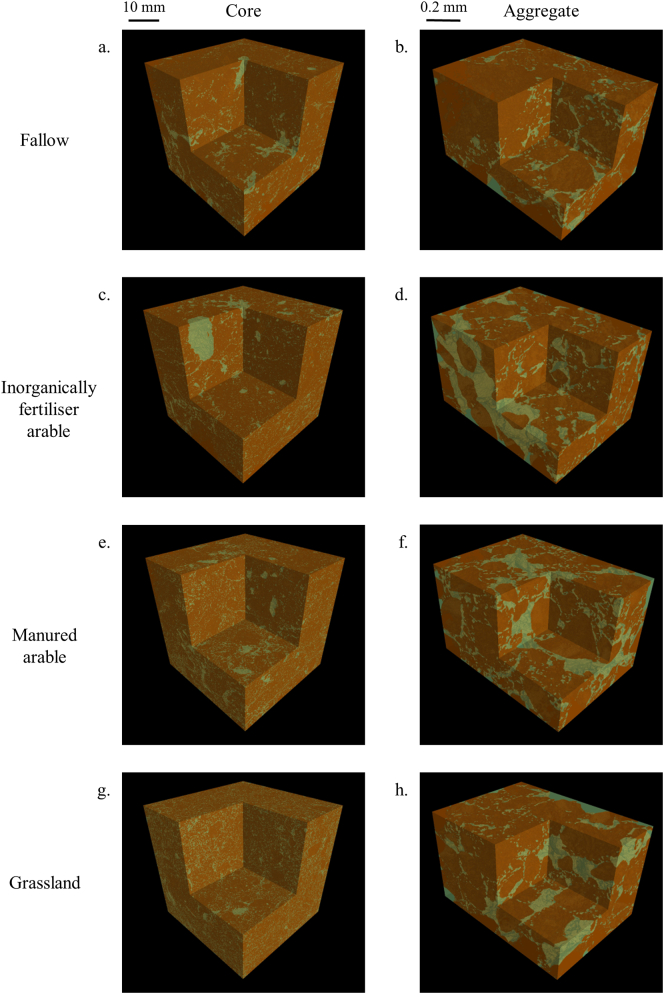
3D representation of sandy soils under different cropping systems visualised at core (40 μm resolution; a, c, e, g) and aggregate (1.5 μm resolution; b, d, f, h) scales, displayed as thresholded images denoting pore (green) or solid (brown) phases. (a, b) bare fallow; (c. d) inorganically fertiliser arable; (e, f) manured arable (g, h) grassland. (For interpretation of the references to colour in this figure legend, the reader is referred to the web version of this article.)

Fig. 3 and A4 illustrate the visualisation of the ROI of the four treatments. In the cores, the presence of stones decreased from bare fallow > arable inorganic equivalent to arable manure > grass (Fig. 3b, A4). The bare fallow and arable inorganic cores appeared relatively porous and contained some large pores (Fig. 3a, c). The arable manure and grassland showed similar types of pores: mostly medium (0.25–1.0 mm) and small pores (<0.25 mm; respectively Fig. 3b, d). There was no significant difference in the proportion of stone for all treatments (Table A2). However, no noticeable difference was observed between the aggregates (Fig. 3, A4).

#### 3.2.2 3D characteristic analysis

Total porosity at the core scale was significantly greater under fallow and inorganically fertilised arable than grassland and manured arable with no significant difference between fallow and inorganically fertilised arable, nor between grassland and manured arable (Table 5). In contrast, at the aggregate scale, there were no significant differences in total porosity between any of the four treatments (Table 5).

**Table 5:** Total porosity in relation to management type at the core (base resolution 40 μm) and aggregate (base resolution 1.5 μm) scale of the sandy soil, expressed as percentage of pores relative to the total volume (mean ± pooled standard error).

Treatment	*n*	Core	Aggregate
Fallow	4	21.1 (±0.98)	23.7 (±0.68)
Inorganically fertilised arable	5	19.6 (±0.87)	24.4 (±0.61)
Manured arable	4	14.6 (±0.98)	24.8 (±0.68)
Grassland	4	13.3 (±0.98)	25.4 (±0.68)
*P*_F_		0.002	<0.001

**Fig. 4:**
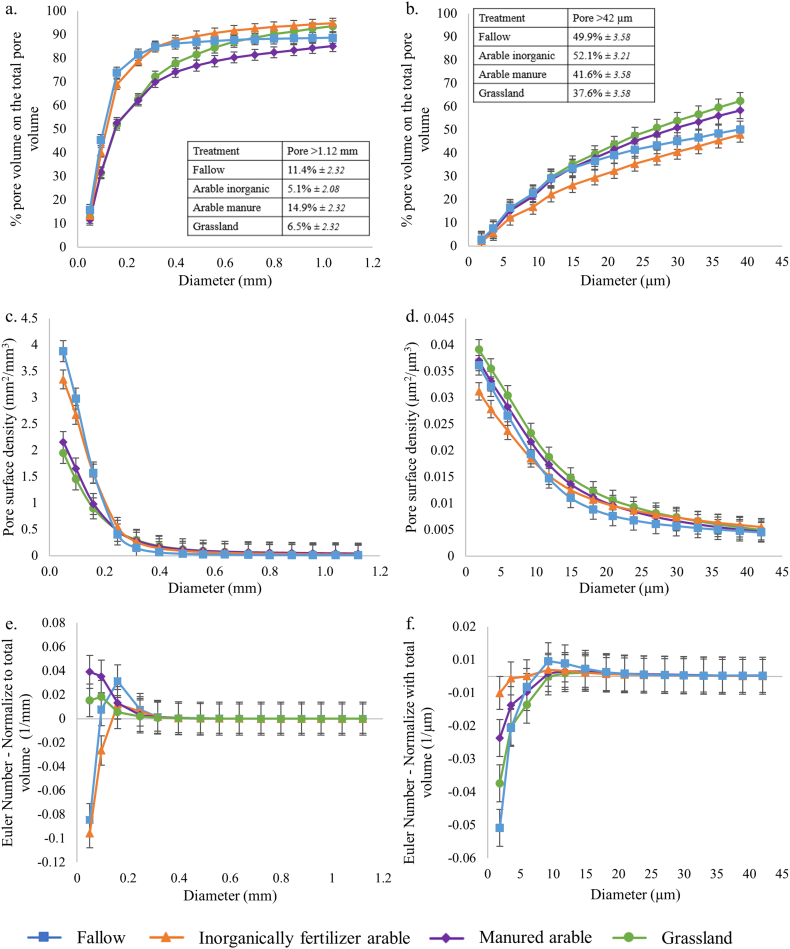
Soil pore characteristics of sandy soils under different cropping systems at core (40 μm resolution; a, c) and aggregate (1.5 μm resolution; b, d, e) scales: (a, b) cumulative pore distribution of cores; (c, d) surface density; (e, f) connectivity. Points indicate means, whiskers denote pooled standard errors.

At the core scale, the pore size distribution normalised to the total ROI volume displayed contrasting behaviours for the pore sizes < 0.25 mm: inorganically fertiliser arable and fallow treatment contained the greatest proportion of the pore sizes compared to grassland and manured arable treatments (Fig. A5a). There was no difference beyond 0.25 mm, except for pores larger than 1.12 mm, here with a greater proportion for fallow and manured arable compared to grassland and inorganically fertiliser arable (Fig. A5a). The nature of the cumulative pore size distributions was different than the pore size distribution normalised to the total ROI and could be classified into three categories: fallow and inorganically fertilised arable, where 70% of the pore sizes were <0.16 mm; manured arable where 70% of the pore sizes were <0.32 mm and had a greater proportion of larger pores; and grassland where 70% of the pore sizes were <0.32 mm and had a larger proportion of pores < 0.64 mm compared to manured arable. The proportion of pores > 1.1 mm ranged from 14.9% (manured arable) to 5.1% (inorganically fertilised arable), with the extremes being significantly different (Fig. 4a). The cumulative pore sizes were characterised by two distinct patterns in relation to treatment associated with fallow, inorganically fertilised arable treatments; and grassland and manured arable. For pore sizes < 0.64 mm, Fig. 4a showed a greater proportion of pore sizes smaller than 0.16 mm under fallow and inorganically fertilised arable than grassland and manured arable (respectively around 70% and 50%). The fallow and inorganically fertilised arable treatments showed a similar cumulative pore size distribution across the size range measured, with very few medium sized pores. The distribution of pore sizes under grassland and manured arable were more diverse, as indicated by greater linearity in abundance across the pore size range. Pore size distributions of grassland and manured arable soils were similar up to a pore size of 0.56 mm and beyond this point, the distribution diverged and there was a significantly greater proportion of the largest pore size under manured arable than grassland (Fig. 4a).

At the aggregate scale, a similar separation of the treatments was observed as for the core scale, although the cumulative pore size distributions were more linear, with only a significant difference for the largest pores under fallow and inorganically fertilised arable, than for grassland and manured arable (Figs. 4b, A5b).

At the core scale, pore surface density profiles also divided into two distinct groupings: fallow and inorganically fertilised arable, and grassland and manured arable. These were significantly greater under fallow and inorganically fertilised arable than grassland and manured arable, both of which were congruent across the size range measured (Fig. 4c). The surface density at the smallest pore size (0.05 mm) was greater under fallow than inorganically fertilised arable, however for the larger pore size (>0.05 mm) the surface density was not significantly different and decreased similarly under both treatments. For pore sizes > 0.25 mm, there were no significant differences between any of four treatments (Fig. 4c). At the aggregate scale, pore surface profiles were similar in their non-linear nature, with inorganically fertilised arable being significantly lower than other treatments over the range < 3.56 μm (treatment x size class *P* < 0.001; Fig. 4d).

At the core scale, the connectivity was significantly greater for fallow and inorganically fertiliser arable compared to grassland and manured arable, with respect to pores < 0.09 mm, and there was no significant differences beyond this (*P* *>* 0.05; Fig. 4e). At the aggregate scale, the values of pore connectivity for all treatments were relatively small, suggesting that the overall pore system for the sandy soil was poorly connected. The connectivity of pores at 1.86 μm was significantly different under all four treatments with a ranking of fallow > grassland > manured arable > inorganically fertilised arable (Fig. 4f). There was then a general trend of decreasing connectivity above this size, with convergence of all treatments for pores > 5.97 μm.

At the core scale, the soil porosity within the VOI (which was used for permeability calculations) was linearly related to the porosity of the entire core (*P* < 0.001), but was on average 22% less (data not shown), again revealing that centimetre-scale pore geometry was highly heterogeneous. Within the VOI, the porosity of manured arable and grassland treatments was similar (mean 17%), as was the case for inorganically fertilised arable and fallow (mean 21%), with the later pair being significantly greater than the former (*P* < 0.01; Table 5). At this scale, modelled permeability was not significantly different between all treatments (Table 6). There was a significant positive power-law relationship between mean porosity and permeability for fallow, arable manure and grassland treatments, which was marginally significant for arable inorganic (Fig. A6a). There was no significant difference between the regression coefficients of the power-law relationship between all treatments (Table 7; overall mean 0.68 ± 0.22). At the aggregate scale, there was a direct linear relationship between the porosity for whole aggregates and the VOI used for modelling (data not shown). There was no difference in total porosity between treatments within the VOI used for modelling permeability (Table 6; overall mean 2.22 ± 0.44). Permeability was not significantly different all treatments (Table 6). At this scale, there was a highly significant positive power-law relationship between porosity and permeability for all treatments (Fig. A6b). The regression coefficient was significantly greater in the case of fallow than all other treatments, and significantly smaller in the case of grassland than all other treatments, with arable and arable manure essentially the same, and median to fallow and grassland (Table 7).

**Table 6:** Total porosity in relation to management type (expressed as percentage of pores relative to the total volume), and modelled saturated permeability, of the volume of interest used for modelling, for the sandy soil at the core and aggregate scale (mean ± pooled standard error).

Treatment	*n*	Core		Aggregate	
		Porosity (%)	Permeability (mm^2^)	Porosity (%)	Permeability (mm^2^)
Fallow	4	20.9 (±1.55)	266 (±59.0)	25.0 (±1.71)	2.44 (±0.45)
Inorganically fertilised arable	5	21.2 (±1.38)	406 (±52.8)	26.0 (±1.71)	2.84 (±0.40)
Manured arable	4	16.4 (±1.55)	501 (±59.0)	24.3 (±1.52)	1.88 (±0.45)
Grassland	4	17.0 (±1.55)	464 (±59.0)	24.6 (±1.71)	1.72 (±0.45)
*P*_F_		0.61	0.37	0.98	0.54

**Table 7:** Linear regression coefficients (mean ± standard error) in relation to management type of log porosity vs. log modelled saturated permeability for the sandy soil, at the core (Fig. A6a) and aggregate scale (Fig. A6b).

Treatment	*n*	Core		Aggregate	
		Coefficient^a^	*P*_uncorr_	Coefficient^a^	*P*_uncorr_
Fallow	12	0.58 (±0.25)	0.04	2.69 (±0.51)	<0.001
Inorganically fertilised arable	15	0.37 (±0.18)	0.06	2.08 (±0.21)	<0.001
Manured arable	12	1.08 (±0.25)	0.002	1.90 (±0.20)	<0.001
Grassland	12	0.70 (±0.22)	0.01	1.37 (±0.13)	<0.001
*P*_F_		0.08		0.02	

a Coefficient of x value in fitted equation.

## 4 Discussion

3D quantification of the soil architecture in terms of pore connectivity, pore surface density and pore size distribution, is essential for linking soil structure to fluid flow, gaseous diffusion and soil functions (Rabot et al., 2018) including metabolism. Our data revealed substantial effects of agricultural practice upon both pore architecture and function from these perspectives.

### 4.1 Effect of management on clay soil

The presence of plants increased the soil porosity, as also shown by Helliwell et al. (2017) albeit in the rhizosphere soil images acquired at 12 μm resolution. Where present, perennial vegetation increased soil porosity at both core and aggregate scales to a greater extent than either annual plants interspersed by tilling or bare fallow. Grassland soils had not been ploughed for at least 150 years and the sampled soil contained a very low number of stones (Fig. 1c, Table A1). The greater proportion of larger pores is most likely to have been induced by the diversity of plant roots and their inputs and the presence of associated soil biota (Pires et al., 2017). The decreased occurrence of stones in the surface 10 cm of grassland soil is likely to be due in part to bioturbation, specifically via the surface-deposition of soil by anecic earthworms, which are known to effectively bury objects to lower soil horizons over time (Table A1; Canti, 2003; Hanson et al., 2009). Regular tillage of the fallow and arable soils would however retain stones in the plough layer.

The pore size distributions were expressed in two ways: normalised to the total ROI volume (Fig. A2) and normalised to the total pore volume (Fig. 2a, b), each highlights different information regarding the pore structure. Normalising to the total ROI volume expressed the absolute proportion of the different pore sizes, whereas normalising to the pore volume shows the relative proportion of each pore sizes to the total porosity. At the core scale, both normalisations showed the same trend: a smaller proportion of pore sizes < 0.25 mm for the arable compared to grassland and fallow and greater proportion of pores > 1.12 mm for grassland and arable treatment compared to the fallow treatment (Fig. 2a, A2a). However, at the aggregate scale, the absolute proportion of the pores < 25 μm was greater for grassland and arable than fallow, while the relative abundance showed a greater proportion for arable and fallow compared to grassland. This difference between absolute and relative abundances might be due to the fact that the porosity observed in the grassland was twofold greater than fallow, so the relative proportion accounted for the difference in porosity. At both scales, grassland soil showed a greater range of pore sizes compared to arable or fallow (Figs 2a, b; Fig. A2) which could be emphasised by a greater presence of organic carbon, fungi and a greater abundance of bacteria in grassland treatment as shown by Hirsch et al. (2009) in a previous study working on the same site. Under the arable and fallow treatments, the frequency of stones was greater than the grassland system (Table A1). The presence of stones could be accounted for as a result of the regular ploughing of the soil over the past 60 years which brought them to the soil surface (Rossi et al., 2013). Recent data from another long-term experiment has shown that no-till arable soils have a significantly lower percent of macro-pores than soils subjected to tilling with a chisel plough (Ambert-Sanchez et al., 2016), which corroborates our observation of the high proportion of large pores under the arable at the core scale. The arable treatments showed fewer larger pores at the aggregate scale which emphasizes that ploughing apparently introduced a greater macro-porosity at the core scale.

At the core scale, grassland systems had a greater surface density for the smallest pores (Fig. 2c), in this circumstance the pore-solid interface was expected to be more accessible to micro-organisms and roots, which would be beneficial for water and nutrient uptake. In contrast, the surface density of all visible pores at the core scale was lowest under arable management which could be due to the mechanical disturbance but also the presence of stones since the morphology of a stone has a smoother edge than a pore. Moreover, the decreased surface density in the arable and fallow treatments can be induced by the loss of the elongated pores due to the impact of the machinery (Pagliaia et al., 2003). At the aggregate scale, treatments involving plants had a greater surface density of all pores than bare fallow soil. However, the surface density values were very low. There were statistically significant differences in surface densities between treatments at the aggregate scale but that the magnitude of the effect was very small.

The volume of interest (VOI) was derived from the region of interest (ROI). VOI was used to model the water saturation of the volume and the ROI was used to calculate the pore characteristics using QuantIm. At the core scale, basic pore characteristics were significantly linearly correlated but absolute values were different. These disparities are likely due to the heterogeneous distribution of the pores within ROI, but given the correlation, it is admissible for comparative purposes to study treatment effects. At the aggregate scale, VOI porosity was congruent with ROI porosity (data not shown).

At both scales, across all clay treatments, permeability generally increased with porosity (Table 3). At the core scale this followed a positive power-law relationship with the exponent varying significantly between treatments (Table 4; Fig. A3a). The increase in permeability, with respect to porosity, increased significantly ranking from fallow < arable < grassland, i.e. there is a substantive effect of the extent of plant presence upon this relationship and the intrinsic ability of the soils to conduct water.

At the aggregate scale, the permeability of the fallow and arable treatments was not significantly different despite the difference in porosity (Table 3). However, under both these treatments, the pore size distribution normalised to the total pore volume was similar for the smaller pores (<9.26 μm) and in greater proportion than grassland (Fig. 2b). Here, the increased proportion of smaller pores appears to reduce permeability. Surprisingly, the differences in grassland and arable treatment permeability (Table 3) were not matched by differences in pore connectivity (Fig. 2e) This suggests that pore size distribution and porosity are better pore characteristic descriptors in our study, which is consistent with observation of Blackwell et al. (1990). This observation complements other studies showing that permeability is dependent upon macro-porosity (Cercioglu et al., 2018) and pore-connectivity at the macroscale (Ball, 1981).

### 4.2 Effect of management on sandy soil

For sandy soil, there was similarity between pore structures derived from grassland (i.e. perennial plants) and manured arable (i.e. annual plants with organic inputs); and between the fallow and inorganically fertilised arable (i.e. annual plants with inorganic inputs; Fig. 4a). This observation is supported by another experiment studying C sequestration over 70 years. In this particular soil, addition of organic manure (38 t ha^−1^ every fifth year) was as efficient at C sequestration as growing 3 years' grass and clover in a 5-year arable rotation (Johnston et al., 2017). At the core scale, the systems involving perennial and annual plants with organic inputs (manure) reduced porosity, surface density and connectivity (Table 5, Fig. 4c, e) but increased the diversity of pore sizes relative to the absence of plants and annual plants with inorganic inputs (Fig. 4a, c). This reduction in porosity was also observed, in the rhizosphere soil, for the growth of tomato plant root systems in sandy loam soil by Helliwell et al. (2017). Furthermore, the increase of the porosity in inorganically fertilised arable and fallow treatments could be due to the conventional tillage which is applied to loosen the soil particles (Arvidsson, 1998). The soil structural characteristics associated with manured arable were similar to the grassland treatment, which supports the notion that addition of organic C helps support arable soil structure. Organic inputs decreased the soil porosity and the connectivity, which could decrease permeability of water and nutrients. Both normalisations of pore size distributions showed the same trend: a greater proportion of pores < 0.25 mm under inorganically fertiliser arable and fallow treatments compared to grassland and manured arable (Fig. 4a, A5a).

At the core scale, VOI porosity was linearly correlated with ROI porosity, but the absolute values were different (data not shown). These differences might be induced by the heterogeneous distribution of the pores within the ROI, however due to the correlation, these values were admissible for the comparison of the treatment effects (Table 7). There were no significant differences amongst treatments for the permeability (Table 7).

At the aggregate scale, the only relative difference was observed for the pore connectivity; however, the Euler numbers were less negative, suggesting the pore systems of all treatments were poorly connected (Fig. 4e). Sandy soil is predominantly composed of larger grains than clay soils. This implies that the aggregate scale may not be optimal to observe differences between treatments in the sandy soil. However, the long-term organic management had been proven to have a greater variability in intra-aggregate spatial pore structure for a fine-loamy soil (Kravchenko et al., 2014). In this study, organic matter inputs increased the presence of both larger (>188 μm) and smaller (<13 μm) pores. The most relevant scale to characterise soil micro-structure is contingent upon the texture (Kravchenko et al., 2014; Peth et al., 2008). This was apparent in our study where management effects were detected at the aggregate scale only in the case of the clay soil.

### 4.3 Contrasting effect of management on both soils

The two soil textures exhibited striking differences in soil structure following application of various long-term managements. In both soils, the grassland (and manured arable for the sandy soil) appeared to influence the porosity at the resolutions considered here, increasing for clay soil and decreasing for the sandy soil. Perennial plant inputs and the addition of manure for the arable treatment appear to affect system porosity in their vicinity, contingent on soil texture. We propose that plants modify soil porosity in their vicinity, improving hydraulic function – for example water retention and flow. Indeed, for both soils, under grassland and manured arable there was a decrease in the proportion of smaller pores, which may lead to an increase in permeability. We found that at the core scale, pore connectivity of the clay soil was not significantly different (Fig.2e) and was significantly greater for the inorganically fertilised arable and the fallow treatments compared to manured arable and grassland treatments. Therefore, carbon inputs from perennial plants and addition of organic carbon apparently decreased the connectivity of the pore system which may recover from disturbance associated with management. This supports the observations of Gregory et al. (2009) who found a positive relationship between organic matter content and the resilience of soils to withstand physical compression. However, at the aggregate scale, the clay soil treatments were significantly different depending on management whereas the sandy aggregates were not significantly different in regard to pore structures between the different managements.

There was a positive power-law relationship between porosity and permeability in nearly all treatments, consistent with Luijendijk and Gleeson (2015); this suggests that soil structure is not random but structured through self-organization (Crawford, 1994). The type of cropping system did not significantly affect this relationship except at the core scale in clay soils and aggregate scale in the sandy soils. In both cases, permeability was much greater relative to porosity under grassland than in fallow, with arable intermediate regardless of increased organic status.

## 5 Conclusions

This study revealed profound but contrasting effects of different agricultural management systems, and in particular the role of plants, on soil structure over the long-term and in the context of two soil textures. For both soil textures, perennial and annual plants (associated with organic input for the sandy soil) increased the diversity of pore sizes. In contrast, the effect of plants on porosity and pore connectivity was markedly different between the two soil textures: for clay soil, plants increased the porosity and the connectivity of the pore system, whereas for sandy soil, plants decreased the porosity and the pore connectivity. Hence the hypothesis that the presence of plants increases porosity requires qualification since plants contributed to soil porosity only in the presence of clay: for sandy soil, the presence of plants reduced porosity and connectivity. Our results confirmed the hypothesis that perennial plants invoked greater structural heterogeneity, manifest as a wider range of pore sizes. This study also showed that addition of manure to arable soil had essentially the same effect as continual perennial plants on the maintenance of the soil structure. Incorporation of organic matter (such as root, organic carbon) can be considered as an agent which assists soil structure to reorganise from the tillage by increasing the diversity of the pore sizes and decreasing porosity. Different crop/management systems create different kinds of soil structure, and for each there are a range of consequences depending on the function under consideration.

These data suggest that management systems generate soil structure differently, conferring to soil structure a variety of functions. The contrasting effects of increased plant presence in the two textures bear an intriguing relationship to what may be considered an optimal configuration of pore architecture in the different circumstances. In the context of a cohesive soil, here clay, plant inputs induced greater porosity, pore-connectivity and permeability, arguably advantageous to plants and the soil biome since it increases water availability via diffuse flow paths. For less cohesive soil, here represented by sand, the presence of plants decreased porosity and connectivity, which likewise is beneficial to the plant and soil biota by increasing the propensity for water storage. The inherent cohesion of the soil may alter a plant's response to its environment in terms of optimising water storage and flow at a system level.

## Appendix A Supplementary data

Supplementary material Image 1
