# Supplementary material for: Effects of cropping systems upon the three-dimensional architecture of soil systems are modulated by texture
Source: Geoderma. 2018 Dec 15;332:73–83. doi: 10.1016/j.geoderma.2018.07.002 (PMC6088510; doi:10.1016/j.geoderma.2018.07.002)
Supplement: Supplementary file 1 — Supplementary material [file mmc1.docx]

Appendices

*Effects of cropping systems upon the three-dimensional architecture of soil systems are modulated by texture*

Aurelie Bacq-Labreuil, John Crawford, Sacha J. Mooney, Andrew L. Neal, Elsy Akkari, Cormac McAuliffe, Xiaoxian Zhang, Marc Redmile-Gordon, Karl Ritz

**Supplementary Materials and Methods A.1.** Modelling the saturated hydraulic conductivity

**Fig. A1.** 3D representation of clay soils under different cropping systems visualised at core (40 μm resolution; a, c, e) and aggregate (1.5 μm resolution; b, d, f) scales, displayed as greyscale images denoting Hounsfield attenuation (darker shades relate to lower attenuation: (a, b) fallow; (c.d) arable; (e, f) grassland.

**Fig. A2.** Volume of pores normalised to the total ROI volume (mm³) (a) at the core scale (40 µm resolution) and (b) at the aggregate (1.5 µm resolution) scale. Points indicate means, whiskers denote pooled standard errors; ● grassland ▲Arable ■ Fallow.

**Fig. A3.** Regression analysis in relation to management type of log porosity vs. log modelled saturated permeability for the clay soil: (a) at the core scale and (b) at the aggregate scale. Doted-line are the linear regression on the log-log values; ● grassland ▲arable ■ fallow.

**Fig. A4.** 3D representation of sandy soils under different cropping systems visualised at core (40 μm resolution; a, c, e, g) and aggregate (1.5 μm resolution; b, d, f, h) scales, displayed as greyscale images denoting Hounsfield attenuation (darker shades relate to lower attenuation: (a, b) fallow; (c. d) inorganically fertiliser arable; (e, f) manured arable (g, h) grassland.

**Fig, A5.** Volume of pores normalised to the total ROI volume (mm³) (a) at the core scale (40 µm resolution) and (b) at the aggregate (1.5 µm resolution) scale. Points indicate means, whiskers denote pooled standard errors; ● grassland ♦ manured arable ▲ inorganically fertilizer Arable ■ Fallow.

**Fig. A6.** Regression analysis in relation to management type of log porosity vs. log modelled saturated permeability for the clay soil: (a) at the core scale and (b) at the aggregate scale. Doted-line are the linear regression on the log-log values; ● grassland ♦ manured arable ▲inorganically fertiliser arable ■ fallow.

**Supplementary Materials and Methods A.1.** Modelling the saturated hydraulic conductivity

The hydraulic conductivity of both core and aggregate samples was calculated numerically by simulating water flow through the pore geometry derived by imagery for each sample as driven by gravity. The flow process was simulated with the lattice Boltzmann model by tracking the movement and collisions of a number of fictitious particles under rules that the collisions conserve mass and momentum. We use the multiple-relaxation time (MRT) (d’Humieres *et al.*, 2002) to describe the propagations of all particle distribution functions as follows:

$f_{i}\left(\text{x}+\delta t\text{e}_{i},t+\delta t\right)=f_{i}\left(\text{x},t\right)+M^{-1}SM\left[f_{i}^{eq}\left(\text{x},t\right)-f_{i}\left(\text{x},t\right)\right],$ (1)

where$f_{i}\left(\text{x},t\right)$is the particle distribution function at location ***x*** and time *t* moving with velocity of ***e****_i_*, δ*x* is the size of the voxels in the image, δt is time step, $f_{i}^{eq}\left(\text{x},t\right)$ is equilibrium distribution function - the value of$f_{i}\left(\text{x},t\right)$at equilibrium, *M* is a transform matrix and *S* is the collision matrix. In Eq. (1), the product M*f* transforms the particle distribution functions to a moment space and the operation $m=SM\left[f_{i}^{eq}\left(\text{x},t\right)-f_{i}\left(\text{x},t\right)\right]$ performs the collision in the moment space. The post-collision results in the moment space are transformed back to particle distribution functions by$M^{-1}m.$ In this paper, we used the D3Q19 lattice in which the particle distribution functions move in 19 directions with 19 velocities (Qian *et al.*, 1992). The collision matrix is diagonal, the terms in which used in this paper are$\begin{matrix} S=\left(s_{0},s_{1},s_{2},s_{3},s_{4},s_{5},s_{6},s_{7},s_{8},s_{9},s_{10},s_{11},s_{12},s_{13},s_{14},s_{15},s_{16},s_{17},s_{18}\right)^{T}, \\ s_{0}=s_{3}=s_{5}=s_{7}=0, \\ s_{1}=s_{2}=s_{9-15}=1/\tau, \\ s_{4}=s_{6}=s_{8}=s_{16-18}=8(2-\tau^{-1})/(8-\tau^{-1}), \end{matrix}$ (2)

The water simulated by the above model has a kinematic viscosity of $\mu=\delta x^{2}(\tau-0.5)/6\delta t$ and pressure of $p=\rho\delta x^{2}/3\delta t^{2}$. The equilibrium moments$m^{eq}=Mf^{eq}$are defined as follows

$\begin{matrix} m_{0}^{eq}=\rho, \\ m_{1}^{eq}=-11\rho+19\left(j_{x}^{2}+j_{y}^{2}+j_{z}^{2}\right)/\rho_{0}, \\ m_{2}^{eq}=3\rho-5.5\left(j_{x}^{2}+j_{y}^{2}+j_{z}^{2}\right)/\rho_{0}, \\ m_{3}^{eq}=j_{x},\text{ }m_{5}^{eq}=j_{y},\text{ }m_{7}^{eq}=j_{z}, \\ m_{4}^{eq}=-2j_{x}/3,\text{ }m_{6}^{eq}=-2j_{y}/3,\text{ }m_{8}^{eq}=-2j_{z}/3, \\ m_{9}^{eq}=\left(2j_{x}^{2}-j_{y}^{2}-j_{z}^{2}\right)/\rho_{0},\text{ }m_{10}^{eq}=\left(2j_{x}^{2}-j_{y}^{2}-j_{z}^{2}\right)/2\rho_{0},\text{ } \\ m_{11}^{eq}=\left(j_{y}^{2}-j_{z}^{2}\right)/\rho_{0},\text{ }m_{12}^{eq}=\left(j_{y}^{2}-j_{z}^{2}\right)/2\rho_{0}, \\ m_{13}^{eq}=j_{x}j_{y}/\rho_{0},\text{ }m_{14}^{eq}=j_{y}j_{z}/\rho_{0},\text{ }m_{15}^{eq}=j_{x}j_{z}/\rho_{0},\text{ } \\ m_{16}^{eq}=m_{17}^{eq}=m_{18}^{eq}=0.\text{ } \\ \text{ } \end{matrix}$ (3)

The density ρ and moment ***j*** of the water are calculated from

$\begin{matrix} \rho=\sum_{i=0}^{18} f_{i}, \\ \text{j}=\rho_{0}\text{u}=\sum_{i=1}^{18} f_{i}\text{e}_{i}, \end{matrix}$ (4)

where ρ_0_ is a reference density to ensure that the water simulated by the above model is incompressible when the flow reaches steady state.

Numerical implementation of the above model on the soil image involves two steps. The first one is to calculate the collision in moment space and then transform the results back to particle distribution function, i.e. to calculate$f_{i}^{*}=f_{i}\left(\text{x},t\right)+M^{-1}SM\left[f_{i}^{eq}\left(\text{x},t\right)-f_{i}\left(\text{x},t\right)\right].$ The second step is to stream the post-collision result$f_{i}^{*}$ to a new location at $\text{x}+\delta t\text{e}_{i}$ over a time period of δ*t*. Whenever$f_{i}^{*}$hits a solid voxel during the streaming step, we used the bounce-back method to solve it by sending$f_{i}^{*}$ back to where it was before the streaming. Such a treatment results in a non-slip boundary at which the water velocity is zero.

For each VOI, we calculating its permeability by maintaining a thin water film on its top and then simulating its flow in the pore geometry driven by gravity. The four sides of the VOI were treated as periodic boundary in which a particle distribution function coming out of the image from one side was sent back into the image through its opposite side by keeping its mass and momentum unchanged. The initial water velocity in the simulations was zero everywhere and once the flow was deemed to have reached steady state, water velocity in all voxels were sampled. The permeability of the VOI was calculated as follows assuming that the average water flow rate across the VOI was proportional to the gravity applied to drive the water flow

$Q=-\frac{k}{u}g,$ (S5)

where *Q* is the average flow rate across the image and *g* is the gravitational acceleration. The permeability *k* is calculated from

$k=\frac{\mu}{Ng}\sum_{i=1}^{N} u_{z}(x_{i}),$ (S6)

where *N* is the number of voxels including the solid voxels and $u_{z}(x_{i})$is the vertical velocity component in the voxel centred at *x_i_*.

References

d’Humieres, D., Ginzburg, I., Krafczyk, M., Lallemand, P. & Luo, L.-S. 2002. Multiple-relaxation-time lattice Boltzmann models in three dimensions. *Philosophical Transactions of the Royal Society A: Mathematical, Physical and Engineering Sciences*, **360**, 437–451, (At: http://rsta.royalsocietypublishing.org/cgi/doi/10.1098/rsta.2001.0955. ).

Qian, Y.H., Dhumieres, D. & Lallemand, P. 1992. Lattice BGK models for Navier-Stokes equation. *Europhysics Letters*, **17**, 479–484.

Table A.1. Quantification of the stones contained in ROI volume (expressed as a percentage of stones relative to ROI volume) for the clay soil at the core scale (mean ± pooled standard error).

| Treatment | n | Proportion of stone (%) |
| --- | --- | --- |
| Fallow | 3 | 15.01 (± 4.89) |
| Arable | 4 | 11.23 (± 4.24) |
| Grassland | 4 | 1.55 (± 4.24) |
| P_F_ |  | 0.039 |

Table A.2. Quantification of the stones contained in ROI volume (expressed as a percentage of stones relative to ROI volume) for the sandy soil at the core scale (mean ± pooled standard error).

| Treatment | n | Proportion of stone (%) |
| --- | --- | --- |
| Fallow | 4 | 3.89 (± 0.94) |
| Inorganically fertiliser arable | 5 | 5.21 (± 0.85) |
| Manured arable | 4 | 5.96 (± 0.94) |
| Grassland | 4 | 3.48 (± 0.94) |
| P_F_ |  | 0.315 |

a.

b.

c.

d.

e.

f.

Core

Aggregate

Fallow

Arable

Grassland

10 mm


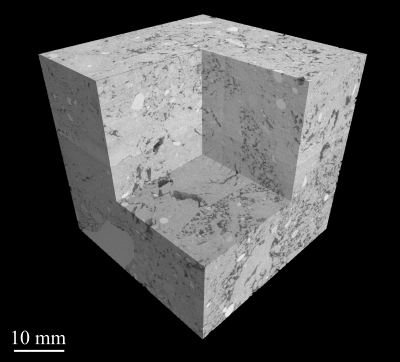

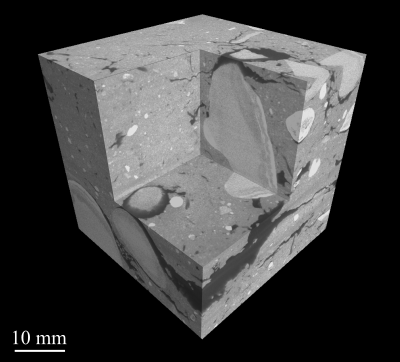

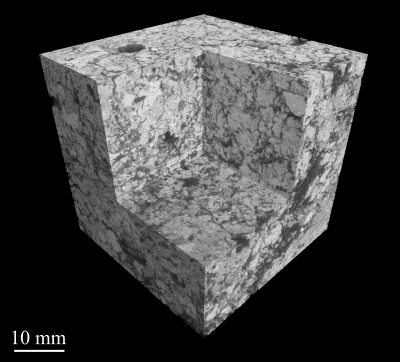

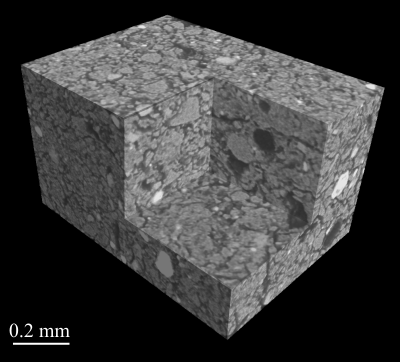

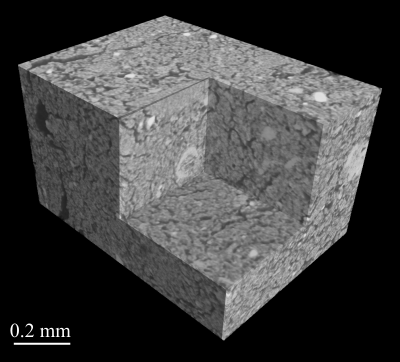

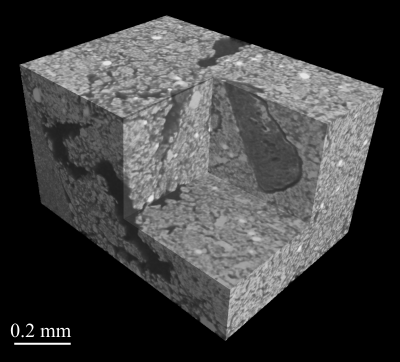


0.2 mm

**Fig. A1.** 3D representation of clay soils under different cropping systems visualised at core (40 μm resolution; a, c, e) and aggregate (1.5 μm resolution; b, d, f) scales, displayed as greyscale images denoting Hounsfield attenuation (darker shades relate to lower attenuation: (a, b) fallow; (c.d) arable; (e, f) grassland.

**
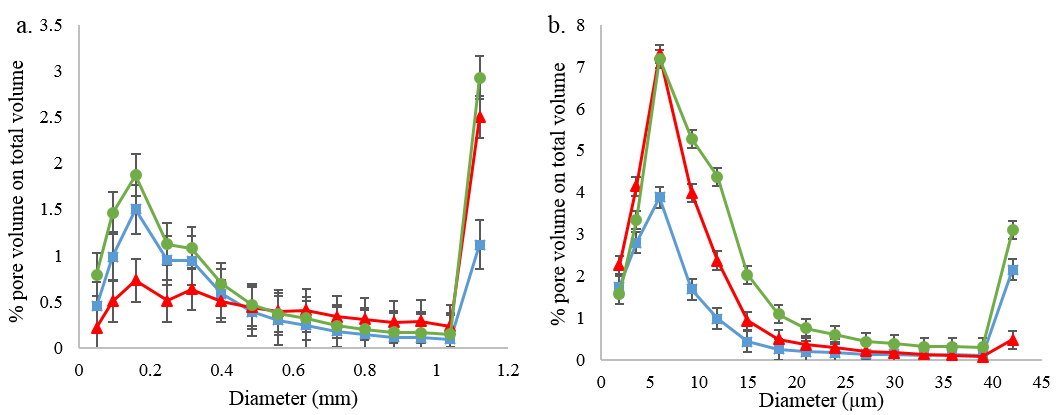
**

**Fig. A2.** Volume of pores normalised to the total ROI volume (mm³) (a) at the core scale (40 µm resolution) and (b) at the aggregate (1.5 µm resolution) scale. Points indicate means, whiskers denote pooled standard errors; ● grassland ▲Arable ■ Fallow.


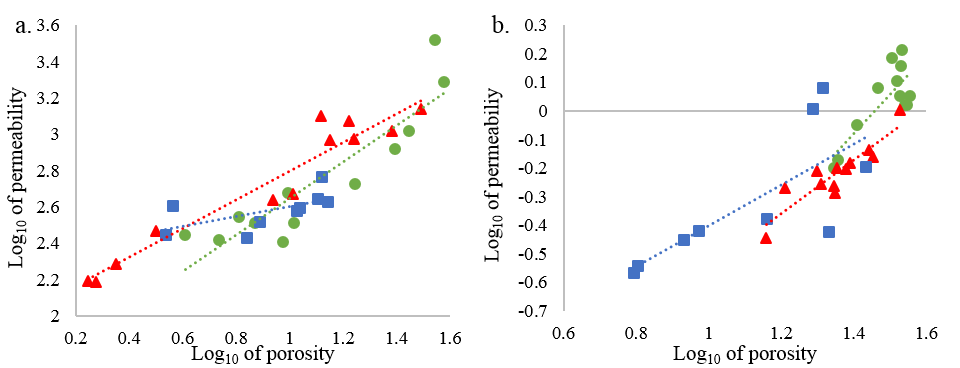


**Fig. A3.** Regression analysis in relation to management type of log porosity vs. log modelled saturated permeability for the clay soil: (a) at the core scale and (b) at the aggregate scale. Doted-line are the linear regression on the log-log values; ● grassland ▲arable ■ fallow.

a.

b.

c.

d.

e.

f.

Core

Aggregate

Fallow

Inorganically fertiliser arable

Manured arable

10 mm

0.2 mm

g.

h.

Grassland


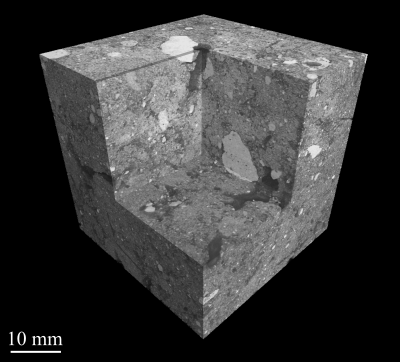

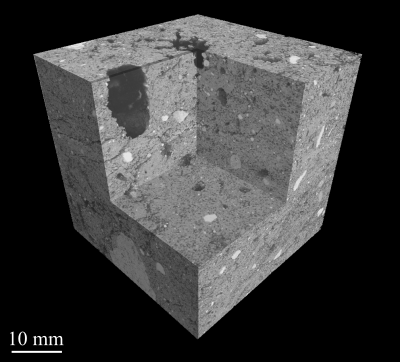

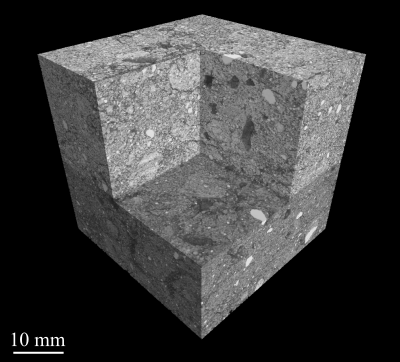

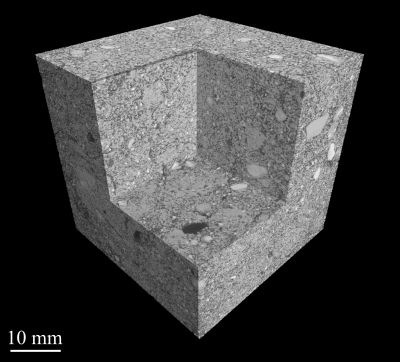

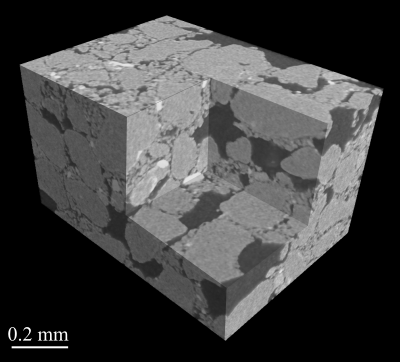

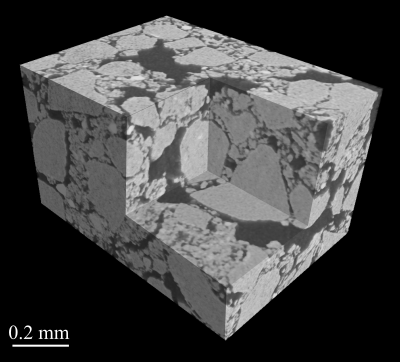

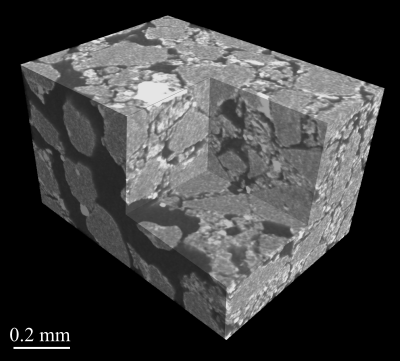

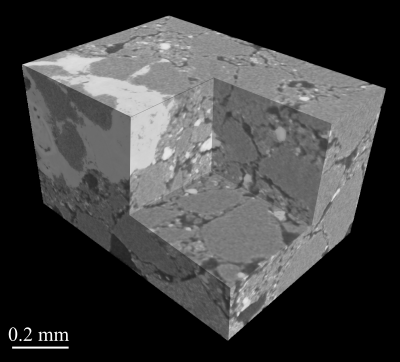


**Fig. A4.** 3D representation of sandy soils under different cropping systems visualised at core (40 μm resolution; a, c, e, g) and aggregate (1.5 μm resolution; b, d, f, h) scales, displayed as greyscale images denoting Hounsfield attenuation (darker shades relate to lower attenuation: (a, b) fallow; (c. d) inorganically fertiliser arable; (e, f) manured arable (g, h) grassland.

**
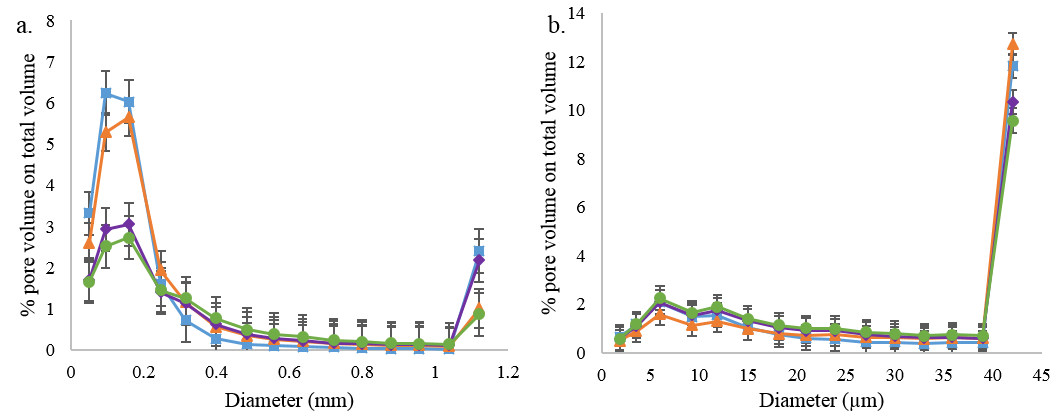
**

**Fig, A5.** Volume of pores normalised to the total ROI volume (mm³) (a) at the core scale (40 µm resolution) and (b) at the aggregate (1.5 µm resolution) scale. Points indicate means, whiskers denote pooled standard errors; ● grassland ♦ manured arable ▲ inorganically fertilizer arable ■ fallow.


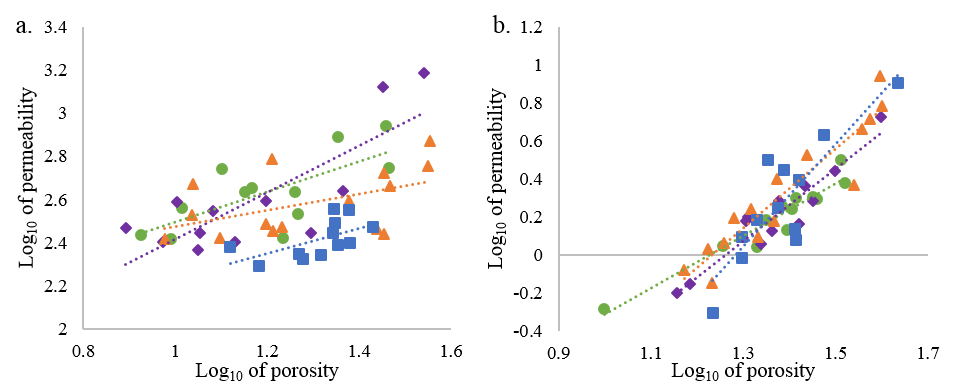


**Fig. A6.** Regression analysis in relation to management type of log porosity vs. log modelled saturated permeability for the clay soil: (a) at the core scale and (b) at the aggregate scale. Doted-line are the linear regression on the log-log values; ● grassland ♦ manured arable ▲ inorganically fertiliser arable ■ fallow.
